# Molecular Changes of Lung Malignancy in HIV Infection

**DOI:** 10.1038/s41598-018-31572-6

**Published:** 2018-09-03

**Authors:** Jianghua Zheng, Lin Wang, Zenghui Cheng, Zenglin Pei, Zhiyong Zhang, Zehuan Li, Xuan Zhang, Dong Yan, Qianlin Xia, Yanling Feng, Yanzheng Song, Weiping Chen, Xiaoyan Zhang, Jianqing Xu, Jin Wang

**Affiliations:** 1 Shanghai Public Health Clinical Center, Fudan University, 2901 Caolang Road, Jinshan District, Shanghai, 201508 P.R. China; 2 Department of Laboratory Medicine, Zhoupu Hospital Affiliated to Shanghai University of Medicine & Health Sciences, Shanghai, 201318 China; 3 Department of Radiology, Ruijin Hospital, School of Medicine, Shanghai Jiaotong University, Shanghai, China; 4 Department of General Surgery, Zhongshan Hospital, Fudan University, 200032 Shanghai, P. R. China; 5 Department of Medical Oncology, Beijing Chaoyang Hospital affiliated to Capital Medical University, Beijing, China; 6 Microarray Core, National Institute of Diabetes and Digestive and Kidney Diseases, National Institutes of Health, Bethesda, MD 20892 USA

## Abstract

Malignancy of the lung is a major source of morbidity and mortality in persons with human immunodeficiency virus infection; as the most prevalent non-acquired immunodeficiency syndrome-defining malignancy, it represents an important and growing problem confronting HIV-infected patients. To evaluate the molecular changes of lung malignancy in HIV infection, we analyzed differential gene expression profiles and screened for early detection biomarkers of HIV-associated lung cancer using Affymetrix arrays and IPA analysis. A total of 59 patients were diagnosed with HIV-associated lung cancer from Jan 2010 to May 2018. The primary outcome was a significant difference in survival outcome between stages III-IV (10.46 ± 1.87 months) and I-II (17.66 ± 2.88 months). We identified 758 differentially expressed genes in HIV-associated lung cancer. The expression levels of SIX1 and TFAP2A are specifically increased in HIV-associated lung cancer and are associated with poorly differentiated tumor tissue. We also found decreased ADH1B, INMT and SYNPO2 mRNA levels in HIV lung cancer. A comprehensive network and pathway analysis of the dysregulated genes revealed that these genes were associated with four network functions and six canonical pathways relevant to the development of HIV-associated lung cancer. The molecular changes in lung malignancy may help screen the growing population of HIV patients who have or will develop this malignancy.

## Introduction

The acquired immunodeficiency syndrome (AIDS) epidemic has had a devastating global impact in the last two decades; millions do not know they are infected with human immunodeficiency virus (HIV) until they develop an opportunistic infection^1^. Patients with HIV/AIDS have a substantially elevated risk of developing Kaposi’s sarcoma, non-Hodgkin’s lymphoma and (in women) cervical carcinoma^2,3^, which are regarded as AIDS-defining malignancies. All malignancies, AIDS-defining and non-AIDS defining, account for up to 1/3 of all deaths in HIV-positive patients^4,5^. Application of highly active antiretroviral therapy (HAART) has deeply changed the landscape of HIV-associated malignancies, and some AIDS-defining tumors have drastically declined. However, an elevated risk has been observed for non-AIDS-defining tumors in HIV-infected people; malignancies of the lung have appeared as a major source of morbidity and mortality in persons with HIV infection^6,7^ and are the third-most common malignancy among HIV-infected persons^8^. Lung cancer is diagnosed when locally advanced or metastatic in most cases, which is similar to patients with unknown HIV status, and adenocarcinoma (AC) is the most common histological subtype^9^. Moreover, some studies from the pre-HAART era also demonstrated an increased risk of lung cancer in HIV-infected patients^10–12^.

All HIV-infected patients with lung malignancy need to undergo staging evaluation earlier in treatment, which will help the chemotherapy for these patients. However, data on the efficacy and toxicity of chemotherapy are few and imprecise^10^. There are several oral agents available for patients who harbor specific mutations, but little is known about mutations and affected pathways in HIV-infected patients with lung cancer^13^. Development of lung cancer in patients with HIV has been linked to various factors, including immunosuppression, CD4 count, and viral load, and cigarette smoking is an important risk factor for lung cancer in HIV patients. Immunosuppression, but not HIV infection, accounts for the higher rates of lung cancer in HIV patients^14^. The HIV tat gene product increases the expression of some proto-oncogenes, including c-myc, c-fos and c-jun. Downregulation of HIV-tat interacting protein promotes metastatic progression of lung cancer^9^. However, there is no clear relationship between the degree of immunosuppression and the risk of lung cancer, so the decision to screen an HIV-infected patient for cancer should include an assessment of individualized risk for cancer, life expectancy, and the harms and benefits associated with the screening test and its potential outcome. Thus, screening the differentially expressed genes in lung cancer with HIV infection needs to be discussed.

Understanding the mechanisms underlying lung carcinogenesis in HIV infection may improve its treatment and the screening of the growing population of HIV patients who have or will develop this malignancy. The aim of this study is to heighten the awareness of lung malignancies occurring in HIV/AIDS while highlighting some of the clinical features in order to facilitate early recognition and diagnosis.

## Results

### Patient characteristics

Among the 59 patients with HIV-associated lung cancer enrolled in the study, the age of patients with lung cancer ranged from 40–77 years, and the average age was 56.40 ± 9.12 years. Fully 88.14% of patients with HIV-associated lung cancer were male, and only 11.86% were female. The pathological types were as follows: adenocarcinoma (36 cases), squamous cell carcinoma (14 cases) and small cell lung cancer (SCLC; 9 cases). The corresponding clinical characteristics of these patients are presented in Table 1. We found that the median overall survival (OS) duration of the 59 patients was 14.12 months (95% CI, 10.63–17.61 months). Although OS did not differ by age, sex, smoking, HAART, complication, CD4^+^ count, or pathological type among these patients by univariate analysis with SPSS, there were significant differences in survival outcome between TMN stages I-II (17.66 ± 2.88 months) and stages III-IV (10.46 ± 1.87 months) (p = 0.026) by pairwise comparison analysis among pathological types of HIV-associated lung cancer.

**Table 1:** Clinicopathological characteristics of the patients with HIV-associated lung cancer.

Clinicopathological characteristic	No. of Patients	Overall Survival	
	n	Months ± SD	P value
**Age (years)**			0.915
<60	37	13.86 ± 1.81	
≥60	22	14.57 ± 3.78	
**Gender**			0.194
Male	52	13.10 ± 1.58	
Female	7	21.43 ± 9.24	
Smoking*			0.479
+	30	1317 ± 2.17	
−	29	15.11 ± 2.87	
**HAART**			0.619
+	39	15.03 ± 2.26	
−	20	12.17 ± 2.85	
**Complication** ^&^			0.209
+	20	17.50 ± 4.29	
−	29	12.34 ± 1.71	
CD4^+^ count			0.324
<200	14	11.46 ± 2.72	
>200	41	15.70 ± 2.34	
**Lung cancer**			0.921
NSCLC	50	13.83 ± 1.64	
SCLC	9	15.00 ± 8.42	
**Pathological type**			0.717
AC	36	14.83 ± 2.08	
SCC	14	11.15 ± 2.29	
SCLC	9	15.00 ± 8.42	
**TMN stage**			0.026
I-II	30	17.66 ± 2.88	
III-IV	29	10.46 ± 1.87	

*Smoking (−): never smoked; ^**&**^Complication (−): patient without tuberculosis/syphilis/hypertension/emphysema/COPD/coronary heart disease.

### Transcriptomic profiles in HIV-associated lung cancer

To identify possible mechanisms of lung malignancies in HIV infection, we performed gene microarray profiling of AIDS patients with lung cancer early diagnosis biomarkers. We collected 7 pairs of tumor/adjacent normal tissue paraffin specimens and 10 pairs of tumor/adjacent normal fresh tissue samples, and we successfully separated 34 HIV with lung cancer tissue and cancer-adjacent RNA samples (17 pairs). A genome-wide analysis of the gene transcripts expressed in the HIV lung cancer tissue samples was performed using an Affymetrix array. We used Partek software for statistical analysis of microarray data. Figure S1 shows the cancer tissue microarray quality of our AIDS patients with lung cancer and that our chips can distinguish cancer tissues and adjacent tissues. Analysis of the microarray hybridization data revealed that 758 genes exhibited more than a 1.5-fold change in expression level (p ≤ 0.05) in the 4 pairs of HIV lung cancer tumor/adjacent normal tissue samples. Further, using the Cancer Genome Atlas (TCGA) data for multiple cancer types analysis of 52 differentially expressed genes (DEGs), which were identified with a 5.0-fold change in expression level (p ≤ 0.05) in HIV-associated lung cancer (Table 2), we identified 10 DEGs (FAT3, NFASC, SLIT2, FMO2, ITGB8, SCARA5, ABCA6, ABCA8, MACC1, and TACC1) that had a high incidence of genetic alterations in lung adenocarcinoma (TCGA-nature 2014, 6.0–21.0%; TCGA-provisional, 5.0–10.0%) and lung squamous cell carcinoma (5.0–18.0%), with an incidence cutoff value of ≥5.0% in the three lung cancer TCGA lists of 929 patients (Table 3). There were also frequent alterations in these genes across other cancer types, including breast invasive carcinoma (2.1–14.0%) and bladder urothelial carcinoma (0.8–16.0%). Our analysis results suggest that alterations in these 10 candidate genes interact in at least a subset of tumors. Since the large-scale sequencing of human cancers can be used to comprehensively discover mutated genes that confer a selective advantage to cancer cells and find genes that drive cancer based on their patterns of mutation in large patient cohorts^15^, we applied these TCGA datasets for driver gene prediction. We demonstarted that a strong tendency of co-occurrences was noted for genetic alterations in these DEGs between ADH1B and FAT3, FAT3 and SLIT2, FIGNL1 and ITGB8, MACC1 and ITGB8, SCARA5 and TACC1, ABCA6 and ABCA8, FMO2 and NFASC, KIAA0895 and ITGB8, MAL and ABCA8 (p < 0.01) (Table S1). Considering the regulatory role of these 10 candidate genes, the underlying mechanisms and cellular consequences of these interactions could be critical for understanding HIV-associated lung cancer pathology.

**Table 2:** The 52 differentially expressed genes in HIV lung cancer.

Probeset ID	Gene Symbol	Gene Title	p-value	Fold-Change	FDR
204304_s_at	PROM1	prominin 1	0.000	41.78	0.026
204653_at	TFAP2A	transcription factor AP-2 alpha (activating enhancer binding protein 2 alpha)	0.003	32.14	0.104
228347_at	SIX1	SIX homeobox 1	0.007	16.34	0.135
202936_s_at	SOX9	SRY (sex determining region Y)-box 9	0.009	12.46	0.145
202935_s_at	SOX9	SRY (sex determining region Y)-box 9	0.006	11.77	0.125
1568574_x_at	SPP1	secreted phosphoprotein 1	0.004	9.89	0.108
214774_x_at	TOX3	TOX high mobility group box family member 3	0.012	9.37	0.159
240303_at	TMC5	transmembrane channel-like 5	0.003	8.83	0.102
201291_s_at	TOP2A	topoisomerase (DNA) II alpha 170 kDa	0.010	8.81	0.150
223748_at	SLC4A11	solute carrier family 4, sodium borate transporter, member 11	0.012	8.67	0.162
205286_at	TFAP2C	transcription factor AP-2 gamma (activating enhancer binding protein 2 gamma)	0.002	7.75	0.090
1552797_s_at	PROM2	prominin 2	0.002	7.70	0.089
228038_at	SOX2	SRY (sex determining region Y)-box 2	0.002	7.34	0.093
215108_x_at	TOX3	TOX high mobility group box family member 3	0.025	7.08	0.204
219787_s_at	ECT2	epithelial cell transforming 2	0.002	6.91	0.089
238689_at	GPR110	G protein-coupled receptor 110	0.030	6.89	0.216
215071_s_at	HIST1H2AC	histone cluster 1, H2ac	0.014	6.77	0.168
205817_at	SIX1	SIX homeobox 1	0.000	6.76	0.053
201417_at	SOX4	SRY (sex determining region Y)-box 4	0.002	6.62	0.093
203744_at	HMGB3	high mobility group box 3	0.002	6.28	0.093
230660_at	SERTAD4	SERTA domain containing 4	0.002	6.26	0.097
226189_at	ITGB8	integrin, beta 8	0.002	6.06	0.094
213424_at	KIAA0895	KIAA0895	0.005	6.01	0.117
216623_x_at	TOX3	TOX high mobility group box family member 3	0.009	5.80	0.145
222843_at	FIGNL1	fidgetin-like 1	0.013	5.67	0.163
201250_s_at	SLC2A1	solute carrier family 2 (facilitated glucose transporter), member 1	0.001	5.59	0.068
223062_s_at	PSAT1	phosphoserine aminotransferase 1	0.011	5.57	0.157
232151_at	MACC1	metastasis associated in colon cancer 1	0.010	5.40	0.150
217901_at	DSG2	desmoglein 2	0.026	5.34	0.207
235170_at	ZNF92	zinc finger protein 92	0.015	5.03	0.171
203865_s_at	ADARB1	adenosine deaminase, RNA-specific, B1	0.001	−5.06	0.084
213438_at	NFASC	neurofascin	0.000	−5.11	0.053
228268_at	FMO2	flavin containing monooxygenase 2 (non-functional)	0.005	−5.11	0.120
219895_at	TMEM255A	transmembrane protein 255A	0.001	−5.13	0.065
212713_at	MFAP4	microfibrillar-associated protein 4	0.004	−5.22	0.115
225660_at	SEMA6A	sema domain, transmembrane domain (TM), and cytoplasmic domain, (semaphorin) 6A	0.005	−5.24	0.123
204777_s_at	MAL	mal, T-cell differentiation protein	0.001	−5.33	0.075
210038_at	PRKCQ	protein kinase C, theta	0.012	−5.35	0.160
219228_at	ZNF331	zinc finger protein 331	0.003	−5.49	0.098
228850_s_at	SLIT2	slit homolog 2 (Drosophila)	0.001	−5.54	0.069
236029_at	FAT3	FAT atypical cadherin 3	0.001	−5.56	0.083
1554547_at	FAM13C	family with sequence similarity 13, member C	0.000	−5.71	0.060
242290_at	TACC1	transforming, acidic coiled-coil containing protein 1	0.001	−5.78	0.071
209763_at	CHRDL1	chordin-like 1	0.004	−5.85	0.117
209220_at	GPC3	glypican 3	0.015	−6.03	0.171
220170_at	FHL5	four and a half LIM domains 5	0.002	−6.26	0.092
238332_at	ANKRD29	ankyrin repeat domain 29	0.007	−6.58	0.131
235849_at	SCARA5	scavenger receptor class A, member 5 (putative)	0.001	−6.66	0.080
243818_at	SFTA1P	surfactant associated 1, pseudogene	0.001	−6.72	0.069
209612_s_at	ADH1B	alcohol dehydrogenase 1B (class I), beta polypeptide	0.006	−6.73	0.124
204719_at	ABCA8	ATP-binding cassette, sub-family A (ABC1), member 8	0.001	−6.83	0.081
201539_s_at	FHL1	four and a half LIM domains 1	0.002	−6.89	0.088
217504_at	ABCA6	ATP-binding cassette, sub-family A (ABC1), member 6	0.003	−6.91	0.103
209613_s_at	ADH1B	alcohol dehydrogenase 1B (class I), beta polypeptide	0.001	−7.11	0.070
211726_s_at	FMO2	flavin containing monooxygenase 2	0.000	−7.24	0.050
229641_at	CCBE1	collagen and calcium binding EGF domains 1	0.002	−7.51	0.097
224061_at	INMT	indolethylamine N-methyltransferase	0.005	−7.54	0.119
225895_at	SYNPO2	synaptopodin 2	0.002	−7.83	0.092

**Table 3:** The Cancer Genome Atlas consortium data on the incidence of genetic alteration^a^ of DEGs in HIV-associated lung cancer by cancer type.

GENE_SYMBOL	Lung adenocarcinoma (n = 230)^b^		Lung adenocarcinoma (n = 522)		Lung squamous cell carcinoma (n = 177)		Breast Invasive Cancer (n = 963)		Bladder urothelial carcinoma (n = 127)	
	n	%	n	%	n	%	n	%	n	%
FAT3	48	21%	50	10%	26	15%	48	5%	13	10%
NFASC	28	12%	46	9%	9	5%	132	14%	5	4%
SLIT2	21	9%	25	5%	19	11%	20	2.10%	1	0.80%
FMO2	21	9%	37	7%	15	8%	96	10%	20	16%
ITGB8	18	8%	30	6%	10	6%	26	2.70%	9	7%
SCARA5	19	8%	31	6%	13	7%	60	6%	7	6%
ABCA6	18	8%	28	5%	14	8%	76	8%	7	6%
ABCA8	17	7%	27	5%	14	8%	87	9%	10	8%
MACC1	13	6%	25	5%	10	6%	22	2.30%	10	8%
TACC1	14	6%	28	5%	32	18%	134	14%	16	13%

^a^Genetic alterations consist of mutations and/or CNV. ^b^Cancer Genome Atlas Research Network, Nature, 2014^15^.

### Quantitative real-time polymerase chain reaction analysis (qRT-PCR) and immunohistochemical (IHC) staining analyses for validation of DEGs in HIV lung cancer

**Figure 1:**
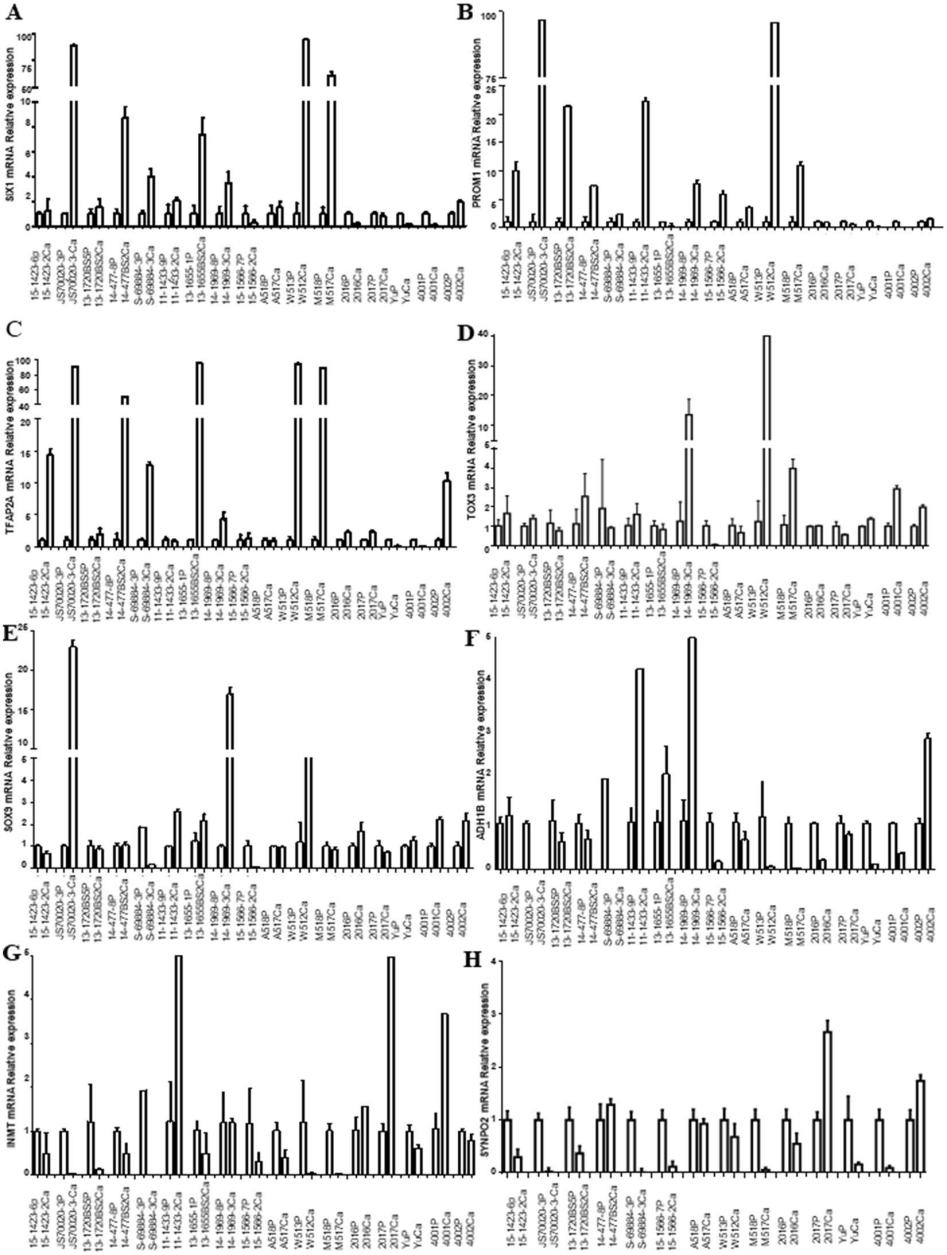
qRT-PCR analysis of SIX1, PROM1, TFAP2A, TOX3, ADH1B, INMT and SYNPO2, mRNA Relative expression in 17 pairs of lung cancer and adjacent non-cancer tissue samples (Cont: adjacent non-cancer tissue; Ca: lung cancer tissue). (**A**) SIX1; (**B**) PROM1; (**C**) TFAP2A; (**D**) TOX3; (**E**) SOX9; (**F**) ADH1B; (**G**) INMT; and (**H**) SYNPO2.

**Figure 2:**
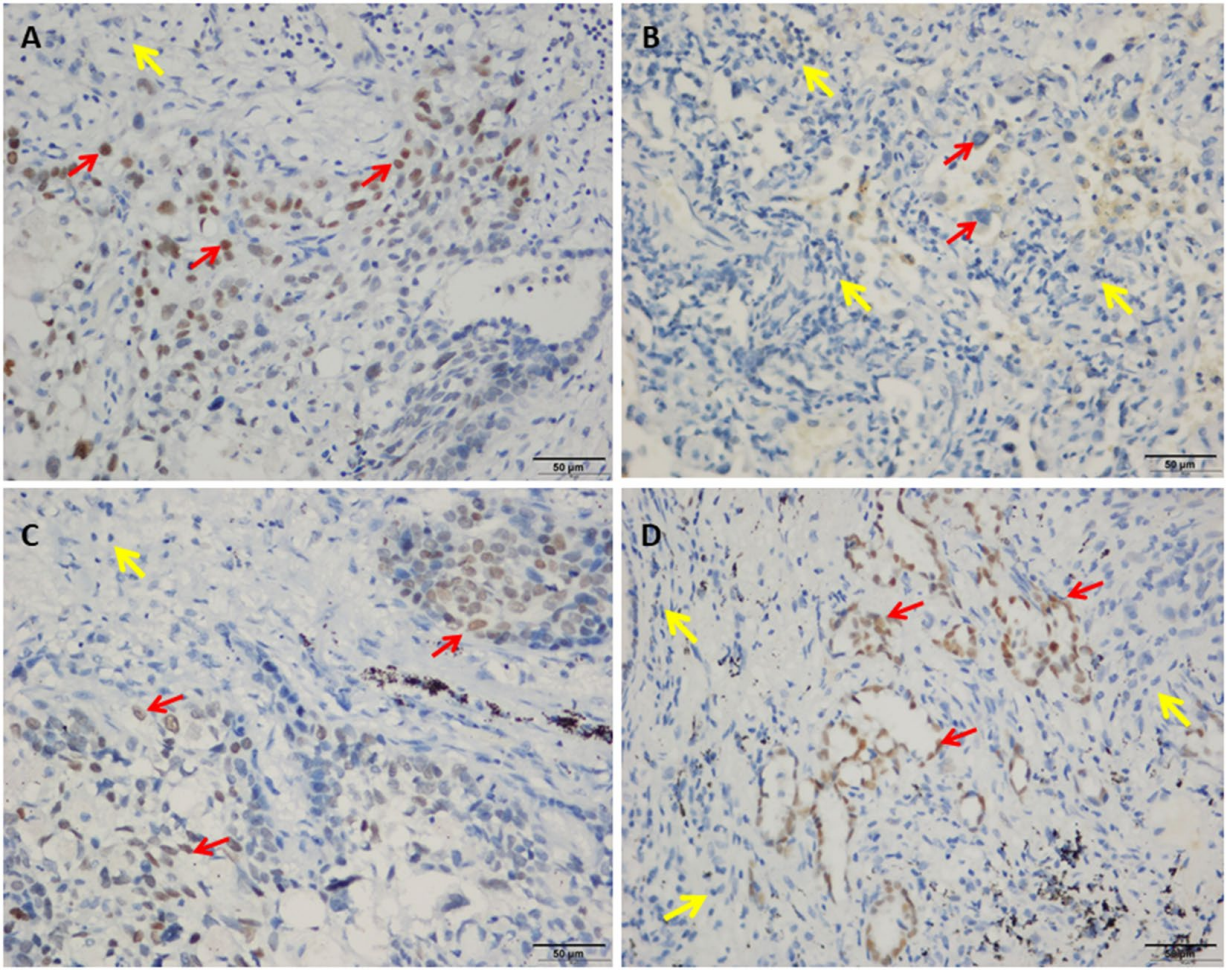
Immunohistochemical analysis of HIV-associated lung tumor tissue samples (squamous cell carcinoma (**A**,**C**) and invasive adenocarcinoma (**B**,**D**) stained with anti-TFAP2A (**A**,**B**) and anti-SIX1 antibodies (**C**,**D**) (original magnification ×400). The normal adjacent lung tissue was labelled with yellow arrows or tumor with red arrows.

**Figure 3:**
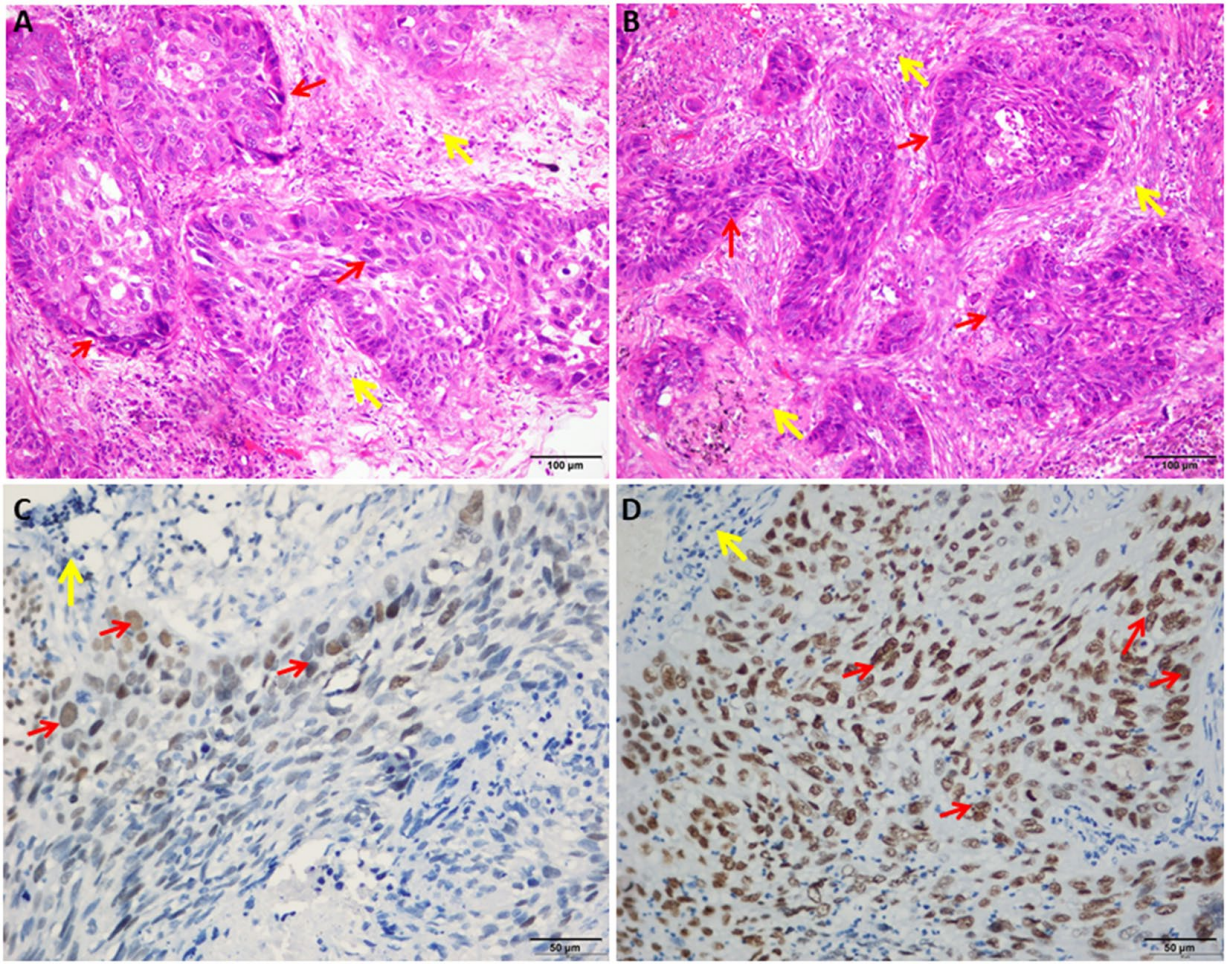
HE staining of lung squamous cell carcinoma tumor tissue samples (Grade II (**A**) and Grade III (**B**) (original magnification ×200). Staining of variably differentiated squamous cell carcinoma (Grade II (**C**) and Grade III (**D**) with an anti-SIX1 (original magnification ×400). The normal adjacent lung tissue was labelled with yellow arrows or tumor with red arrows.

**Figure 4:**
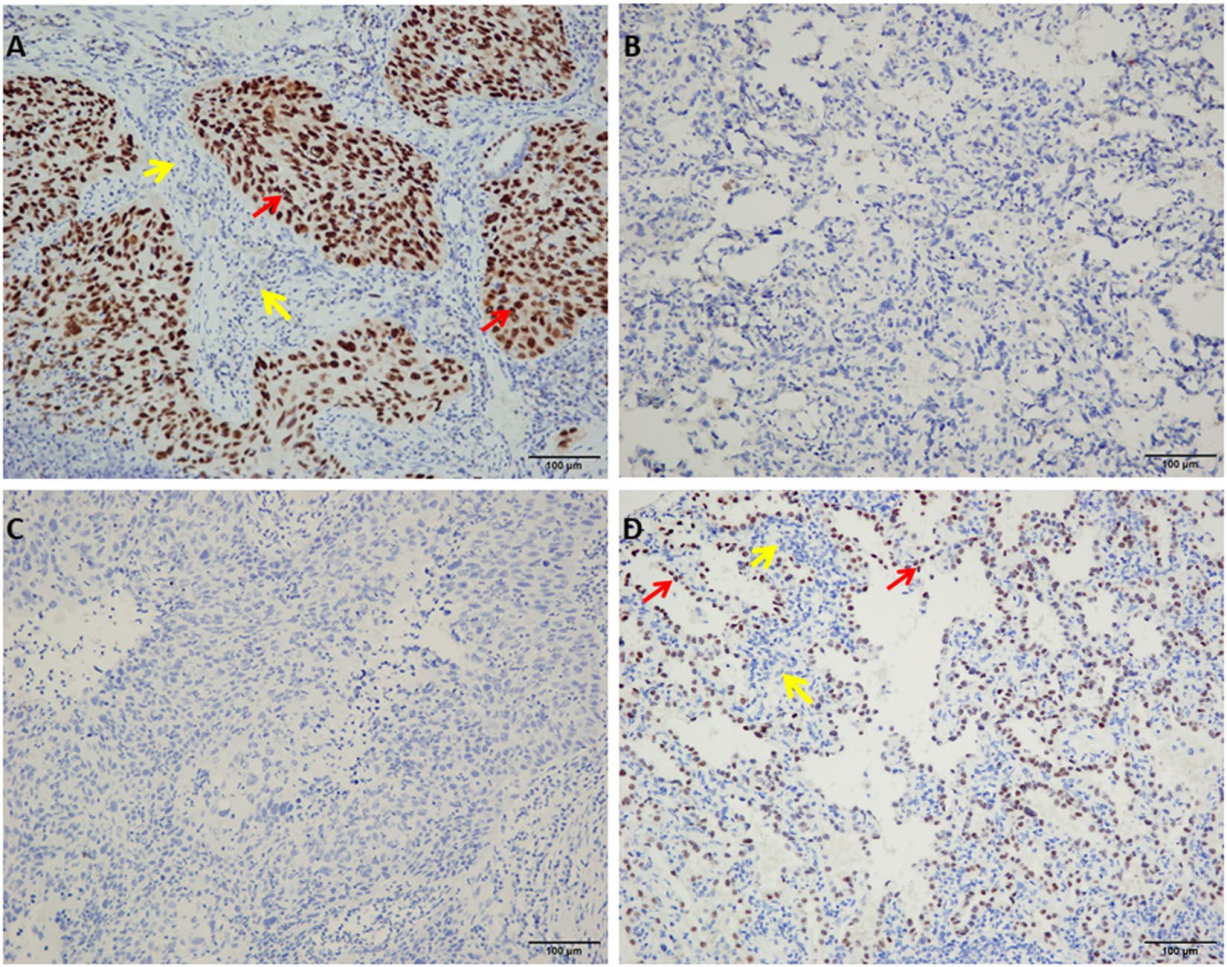
Immunohistochemical analysis of HIV-associated lung tumor tissue samples (squamous cell carcinoma (**A**,**C**) and invasive adenocarcinoma (**B**,**D**) stained with anti-p63 (**A**,**B**) anti-TFF-1 antibodies (**C**,**D**) (original magnification ×200). The normal adjacent lung tissue was labelled with yellow arrows or tumor with red arrows.

qRT-PCR analysis of SIX1, PROM1, TFAP2A, TOX3, SOX9, ADH1B, INMT and SYNPO2, mRNA expression in 17 pairs of lung cancer and adjacent non-cancer tissue samples revealed that 12 of 17 (70.6%) tumors had increased SIX1 mRNA (14.83-fold) or PROM1 mRNA (15.16-fold), 13 of 17 (76.5%) had increased TFAP2A mRNA (26.18-fold), and 8 of 17 (47.1%) tumors had increased TOX3 mRNA (4.54-fold) or SOX9 mRNA (3.79-fold) expression in HIV-associated lung cancer compared to adjacent non-cancer tissue (Fig. 1). At the same time, we also found that 11 of 17 (64.7%) tumors had decreased ADH1B mRNA (20.93-fold) or INMT mRNA (10.22-fold) in HIV lung cancer. Also, 11 of 14 (78.6%) tumors had decreased SYNPO2 mRNA (13.26-fold) in HIV lung cancer, except SYNPO2 was undetectable in 3 pairs of lung cancer and adjacent non-cancer tissue samples (Fig. 1). To investigate the expression status of TFAP2A and SIX1 proteins in benign and malignant lung tissue, we performed IHC staining of 7 lung tumor samples and 7 adjacent non-cancer tissue samples. TFAP2A- and SIX1- specific staining was clearly observed in the nucleus of the lung cancer cells including HIV associated squamous cell carcinoma (SCC) and AC (Fig. 2). For TFAP2A (Fig. 2A,B), a total of 4 of 7 malignant cases showed positive staining for TFAP2A, and 2 of 7 adjacent non-cancer tissue samples were positive. It is interesting that a total of 6 of 7 (85.7%) malignant cases showed positive staining for SIX1, and not one of the adjacent non-cancer tissue samples was positive; the difference in SIX1 expression between lung cancer tissue and adjacent non-cancer tissue samples was statistically significant (p < 0.001) (Table S2). Although there was not different expression of SIX1 between Grade II and Grade III of HIV associated AC (Supplemental Fig. S2), the staining of HIV associated SCC with an anti-SIX1 was variable with poorly differentiated tumor tissue (Grade III) showing higher expression of the protein (Fig. 3). Little or no staining of SIX1 was observed in adjacent non-cancer tissue samples. All these results indicate that the expression levels of SIX1 and TFAP2A are specifically increased in HIV-associated lung cancer. To better distinguish the cancer tissue from the surrounding tissue, we finally stained with p63 and TTF-1 as lung cancer cell specific markers^16^ and found that all the two SCC cases showed strong positive staining for p63 (Fig. 4A), and not one of adenocarcinomas samples was positive (Fig. 4B), but TTF-1 staining was observed in 60.0% of adenocarcinomas (Fig. 4D and Table S2).

### Functional networks and pathways of HIV lung cancer

The genetic networks and cellular pathways dysregulated in HIV lung cancer were identified using the IPA software program. Expression microarray profiling studies revealed that 758 genes were dysregulated in HIV lung cancer. A comprehensive network and pathway analysis of the DEGs revealed that these genes were associated with four network functions and six canonical pathways relevant to the development of HIV lung cancer. The scores take into account the number of focus proteins and the size of the network to approximate the relevance of the network to the original list of focus proteins. In each of the four genetic networks, the DEGs constituted about half of the molecules involved in network-associated (1) cancer, connective tissue disorders, organismal injury and abnormalities; (2) nucleic acid metabolism, small molecule biochemistry, organ morphology; (3) cardiovascular system development and function, embryonic development, organismal development; (4) cancer, organismal injury and abnormalities, developmental disorder (Table S3 and Supplemental Fig. S3). The differentially expressed genes belong to six canonical signaling pathways, such as cellular effects of sildenafil; dopamine-DARPP32 feedback in cAMP signaling; purine nucleotides de novo biosynthesis II; 5-aminoimidazole ribonucleotide biosynthesis I; and tetrahydrofolate salvage from 5,10-methenyltetrahydrofolate pathways (Table S4).

## Discussion

Lung cancer is the most common cause of cancer mortality worldwide for both men and women, and it also represents an important and growing problem confronting HIV-infected patients^5^. The incidence of lung cancer has risen among women in the past several decades, but the incidence is still higher among men than among women^17,18^. Although several mechanisms such as HAART, repeated lung infections, chronic pulmonary inflammation and/or immunosuppression have been reported to promote the development of lung cancer^9,10^, in this study, we found that most patients did not have a low CD4^+^ cell count, with a median CD4^+^ count at lung cancer diagnosis of 281/ml; there were 14 patients with CD4^+^ count <200/mL and 41 patients with CD4^+^ count >200/mL. Moreover, for patients with low CD4^+^ count, no significant differences in survival outcome were seen between CD4^+^ count <200/mL (11.46 ± 2.72 months) and CD4^+^ count >200/mL (15.70 ± 2.34 months) (p = 0.324), which demonstrated that HIV^+^ patients with lung cancer did not seem to be particularly immunosuppressed. We have already identified significant differences in survival outcome between TMN stages I-II (17.66 ± 2.88 months) and stages III-IV (10.46 ± 1.87 months). Lung cancer can often be asymptomatic in the early stages and may be diagnosed purely by chance^19^. Brock found that advanced stage in HIV-infected lung cancer patients was associated with worse survival compared to HIV-uninfected patients^20^. Although our data showed that HIV-infected lung cancer patients have shortened survival mainly due to advanced stage, there is concern for lead-time bias in lung cancer screens^19^. Lead-time bias cannot be excluded as a reason for the longer overall survival in those HIV-infected patients diagnosed as stage I-II compared to stage III-IV. These results implied that TNM was not only a major prognostic factor in the general population but also in HIV and might yield a survival benefit, although our study population was small.

To identify the molecular mechanisms underlying the gene expression profiles of lung malignancy in HIV infection, we performed an integrative network analysis using IPA. Using this tool, we identified four significant networks of lung malignancy in HIV infection (score ≥32; Table S3 and Supplemental Fig. S3). The scores take into account the number of focus proteins and the size of the network to approximate the relevance of the network to the original list of focus proteins. The networks altered in lung malignancy in HIV infection were associated with “Cancer”, “Nucleic acid metabolism”, “Organismal injury” or “Developmental disorder”. We then carried out a canonical pathway analysis of these dysregulated genes in this malignancy and revealed cellular effects of sildenafil; dopamine-DARPP32 feedback in cAMP signaling; purine nucleotides de novo biosynthesis II; 5-aminoimidazole ribonucleotide biosynthesis I; and tetrahydrofolate salvage from 5,10-methenyltetrahydrofolate. Most of these pathways are involved in cellular metabolism and are frequently dysregulated in HIV-associated lung cancer^21–25^. Sildenafil, as an inhibitor of cGMP-degrading phosphodiesterase 5, is used to treat erectile dysfunction and potentiates a cGMP-dependent pathway to promote melanoma growth, which can also affect the innate and adaptive immune system in patients^26,27^.

In the initial biomarker identification stage, gene expression profiling was performed to assay the differentially expressed genes. Validation of the candidate differentially expressed genes was then performed using quantitative real-time polymerase chain reaction assay and immunohistochemistry analysis. We found that SIX1, PROM1, and TFAP2A were upregulated in HIV-associated lung cancer. SIX1 (SIX Homeobox 1) was reported to play a role in the development of tumors, including breast, colorectal, gastric, and pancreatic cancer^28–31^. The SIX family homeobox genes have been demonstrated to be involved in tumor initiation and progression; they play distinct roles in the tumorigenesis of non-small cell lung cancer (NSCLC) and can be potential biomarkers in predicting prognosis of NSCLC patients^32^. SIX1 can promote cell proliferation via reactivating the cell cycle-related proteins cyclin A^33^ and stimulate malignant transformation of nontumorigenic cells^31^. Gene Expression Omnibus database analysis also confirmed that luminal breast cancer patients with SIX1 overexpression had worse overall survival, shorter relapse-free survival, and much worse prognosis^34^. More importantly, it was found to be closely linked to poor clinical prognosis in cancer patients^35^. TFAP2A (transcription factor AP-2 alpha, AP-2α) is a eukaryotic transcriptional factor. The AP-2 family of transcription factors plays a pivotal role in normal development and morphogenesis during embryogenesis^36^. AP-2α expression was positively associated with chemosensitivity in bladder, breast, endometrium, and pancreas cancers, and it was reported to be a predictive marker for good response and survival after cisplatin-containing chemotherapy in several cancers^37–39^. High levels of AP-2α protein in bladder cancer were associated with good response to cisplatin^38^, and it was a newly identified prognostic marker for chemotherapy^40^. As a cancer stem cell marker, PROM1 was identified as both a hematopoietic and neuroepithelial stem cell marker^41,42^. PROM1 has been identified in colorectal, hepatocellular, and pancreatic cancer^43^, as one of the most important markers of tumor-initiating cells and an adverse prognostic factor in colon cancer, gliomas, and medulloblastoma^43,44^, and is associated with decreased survival in a variety of human tumors, including brain, colorectum, endometrium, gliomas, liver, medulloblastoma, NSCLC, ovary, and stomach^45,46^. On the other hand, SYNPO2, ADH1B and INMT were found to be repressed in HIV-associated lung cancer. SYNPO2 (synaptopodin-2) is a repressor of tumor cell invasion, being predominant in prostate acinar epithelial and basal cells, and induces formation of complex stress fiber networks in the cell body^47^. Kopantzev also comfirmed that ADH1B and INMT were down-regulated in NSCLC as compared to adjacent normal tissues using qRT-PCR and microarray analyses^48^. Against this background, there is an opportunity to develop novel gene signatures for HIV-associated cancer, which is good for early detection of HIV-associated lung cancer. These molecular changes of lung malignancy can offer the hope of early detection as well as tracking disease progression and recurrence. Therefore, these cancer biomarkers have provided great opportunities for improving the management of cancer patients by enhancing the efficiency of early detection, diagnosis, and treatment. Finally, we have also realized that our study is exploratory research on the molecular changes of lung malignancy in HIV infection, and the current sample size is small. We hope our molecular insights can optimize cancer screening and prevention strategies for HIV-infected populations and guide the treatment of HIV-associated lung cancer.

## Materials and Methods

### Patients and tissue specimens

This prospective study was approved by the Shanghai Public Health Clinical Center Institutional Review Board. HIV patients who warranted evaluation by computed tomography-guided percutaneous needle biopsy of the lung, cytologic analysis for the evaluation of pleural effusion, and later were histologically diagnosed with lung cancer were enrolled. A total of 59 patients were diagnosed with HIV-associated lung cancer from Jan 2010 to May 2018. The age of the 59 patients with HIV-associated lung cancer varied from 40 to 77 years (median, 56 years), and the clinicopathological features of the patients included age at diagnosis, gender, cigarette smoking, complications, HAART, CD4^+^ count, and TMN stage. Written informed consent was obtained from all patients for use of the tissue samples and clinical records. The study protocol was performed under approval by the Ethic Committee of Shanghai Public Health Clinical Center and all methods were performed in accordance with the relevant guidelines and regulations. All cases were evaluated by two staff pathologists (Y.F., and J.Z.) who were blinded to the clinical outcome.

### RNA purification and Transcriptomic profiles

RNA was purified from HIV-associated lung cancer tumor/adjacent normal tissue samples using Trizol LS reagent (Invitrogen, Carlsbad, CA, USA) and the RNeasy mini kit (Qiagen, Valencia, CA, USA). The quality of the purified RNA was assessed using an Agilent 2100 bioanalyzer (Agilent Technologies, Waldbronn, DE, USA). All the tissue samples came from Shanghai Public Health Clinical Center, Fudan University (Shanghai, China). RNA for transcriptomic profile analysis in the HIV lung cancer tumor tissue samples was from 4 pairs of tumor/adjacent normal tissue samples. After quality assessment using the Agilent NanoChip Bioanalyzer assay, microarray analyses were performed using 2 μg total RNA from each sample with one cycle of complementary RNA amplification according to the Affymetrix (Santa Clara, CA) protocol. The complete microarray datasets have been available on the NCBI Gene Expression Omnibus (GEO) Accession Number: GSE 106937.

### Microarray data analysis and pathway analysis

The subsequent gene lists and associated expression values were uploaded into Partek Pro 6.0 software (Partek, MO), and expression levels were clustered and displayed by GeneSpring 7.3 (Silicon). When appropriate, fold change was calculated as the ratio of the mean of gene expression measures in HIV-associated lung cancer and adjacent non-cancer tissue samples gene expression measures. To determine the potential specific pathways based on changes in gene expression, we used the Ingenuity Pathway Analysis (IPA) software program (Ingenuity, Redwood City, CA) as described previously^49^.

### cBioPortal analysis of the Cancer Genome Atlas data on multiple types of cancer to determine the probability of gene expression alteration

We investigated our candidate genes in the TCGA data via cBioPortal^50^ and generated the probability of alteration of differentially expressed genes for four different types of cancer: lung adenocarcinoma (TCGA nature 2014, n = 230)^51^. TCGA, Provisional, n = 522), lung squamous cell carcinoma (n = 177), breast invasive carcinoma (n = 963), and bladder urothelial carcinoma (n = 127).

### Quantitative real-time polymerase chain reaction analysis

Thirty-four RNA samples were used for qRT-PCR analysis in the HIV lung cancer tumor tissue samples from 13 patients with AC, 3 patients with SCC, and 1 patient with neuroendocrine tumors (NETs) including 7 pairs of tumor/adjacent normal tissue paraffin specimens and 10 pairs of tumor/adjacent normal fresh tissue samples. Total RNA (1 μg) was reverse transcribed to cDNA using the First Strand cDNA synthesis kit (Invitrogen). qRT-PCR was performed using the ABI Prism 7900 HT sequence detection system and Taqman Universal PCR master mix (both from Applied Biosystems, Foster City, CA, USA). Seventeen pairs of lung cancer and adjacent non-cancer tissue samples were used for qRT-PCR analysis. Relative expression of the mRNAs was calculated utilizing the comparative Ct (2^−ΔΔCt^) method with 18S as the endogenous control to normalize the data.

### Immunohistochemical staining

IHC staining for TFAP2A, TTF-1, p63 and SIX1 was performed in 14 formalin-fixed, paraffin-embedded tissue samples from the Shanghai Public Health Clinical Center. The study was approved by the Institutional Review Boards. Paraffin-embedded tissues were dewaxed in xylene, rehydrated in serial concentrations of ethanol, and then rinsed in PBS followed by treatment with 3% H_2_O_2_ to inhibit endogenous peroxidase. After being heated at 60 °C overnight, the sections were incubated with 10% normal goat serum at room temperature for 10 min to block non-specific reactions. This was followed by a PBS wash and incubation with Anti-TFAP2A (Abcam, ab108311), Anti-TTF-1 (Dako Corporation, CA), Anti-p63 (Novocastra Laboratories Ltd, UK), or Anti-SIX1 (Cell Signaling Technology, #16960, Danvers, MA) antibodies for IHC analysis. Fourteen paraffin-embedded tissues were retrieved for IHC analysis. SIX1, TTF-1, p63 and TFAP2A proteins appeared as brownish granules after staining. The expression status of SIX1, TTF-1, p63 and TFAP2A was scored using a 5-point scale based on the intensity of positive staining and the distribution of positive cells under 5 random high-power fields.

### Statistical analysis

Data analyses were performed using SPSS statistical package 17.0 (SPSS Inc., Chicago, IL, USA). Statistical values are presented as the mean ± standard deviation. The Student *t*-test was used to assess differences between groups. A univariate analysis was performed using the Kaplan-Meier estimator method and a log-rank test. The median survival time was calculated using SPSS. p < 0.05 was considered to indicate a statistically significant difference.

## Electronic supplementary material

Supplemental information
