# Supplementary material for: Molecular Changes of Lung Malignancy in HIV Infection
Source: Sci Rep. 2018 Sep 3;8:13128. doi: 10.1038/s41598-018-31572-6 (PMC6120915; doi:10.1038/s41598-018-31572-6)
Supplement: Supplementary file 1 — Supplemental information [file 41598_2018_31572_MOESM1_ESM.pdf]

Supplementary Tables and Figures:

Molecular Changes of Lung Malignancy in HIV Infection

Jianghua Zheng[1, 2,#], Lin Wang[1, #], Zenghui Cheng[1,3, #], Zenglin Pei[1, #], Zhiyong Zhang[1,#], Zehuan Li[1, 4], Xuan Zhang[1], Dong Yan[5], Qianlin Xia[1], Yanling Feng[1], Yanzheng Song[1], Weiping Chen[6], Xiaoyan Zhang[1], Jianqing Xu[1], Jin Wang[1, *]

[1]Shanghai Public Health Clinical Center, Fudan University, 2901 Caolang Road, Jinshan District, Shanghai 201508, P.R. China; [2]Department of Laboratory Medicine, Zhoupu Hospital Affiliated to Shanghai University of Medicine & Health Sciences, Shanghai 201318, China; [3]Department of Radiology, Ruijin Hospital, School of Medicine, Shanghai Jiaotong University, Shanghai, China; [4]Department of General Surgery, Zhongshan Hospital, Fudan University, 200032, Shanghai, P. R. China; [5]Department of Medical Oncology, Beijing Chaoyang Hospital affiliated to Capital Medical University, Beijing, China; [6]Microarray Core, National Institute of Diabetes and Digestive and Kidney Diseases, National Institutes of Health, Bethesda, MD 20892, USA.

Running Title: Molecular Changes of Lung Malignancies in HIV Infection.

***Correspondence should be addressed to:**

Jin Wang, Ph.D.
Shanghai Public Health Clinical Center,
Fudan University,
2901 Caolang Road, Jinshan District,
Shanghai 201508, China;
Ph: 86-21-57036495;
Fax: 86-21-57247094
Email: wangjin@shaphc.org; wjincityu@yahoo.com

[#] These authors contributed equally to this work

1. Supplementary Tables:

1). Supplemental Table S1. Patterns of co-occurrence of DEG alterations in HIV-associated lung cancer in The Cancer Genome Atlas consortium for lung adenocarcinoma (TCGA, Provisional) (n=522).

**DEG**	**Driver gene[c]**	**p value**
ADH1B	FAT3	0.004
FAT3	SLIT2	<0.001
FIGNL1	ITGB8	<0.001
MACC1	ITGB8	<0.001*
SCARA5	TACC1	<0.001
ABCA6	ABCA8	<0.001
FMO2	NFASC	<0.001*
KIAA0895	ITGB8	<0.001
MAL	ABCA8	0.008

[c]Driver genes are those with the highest incidence of alterations in a given dataset. *Strong tendency toward co-occurrence and log odds ratio > 3. TCGA data obtained through cBioPortal.

2). Supplemental Table S2. qRT-PCR and IHC analysis of the differently expressed genes in HIV-associated lung cancer.

Patient/Slide ID	TFAP2A QPCR	TFAP2 A IHC*	SIX1 QPCR	SIX1 IHC*	p63 IHC	TTF-1 IHC	Tumor grade (HE Staining analysis)
15-1423-6P	1.00	-	1.00	-	-	-	-
15-1423-2Ca	14.37	-	1.24	±	-	+	Invasive adenocarcinoma, mostly micro-papillary growth, local glandular carcinoma, grade III.
14-477-8P	1.00	-	1.00	-	-	-	Normal acinar with few alveolar macrophages.
14-477BS2Ca	50.66	-	8.71	+	-	-	Invasive adenocarcinoma, mostly mucinous adenocarcinomas (MAC), local micro-papillary adenocarcinoma, grade II-III.
14-1969-8P	1.00	-	1.00	-	-	-	-
14-1969-3Ca	4.26	+	3.50	-	-	-	Invasive mucinous adenocarcinoma with few Signet ring cell carcinoma (SRCC), grade III.
15-1566-7P	1.00	-	1.00	-	-	-	Congestion, edema, bleeding, infiltration of inflammatory cells.
15-1566-2Ca	1.26	±	0.27	+	-	++	Invasive adenocarcinoma, mostly acinar adenocarcinoma, grade II.
13-1655-1P	1.00	-	1.00	-	-	-	Fibrotic zone
13-1655BS2Ca	95.42	++	7.38	+	+++	-	Squamous cell carcinoma, grade II
13-1720BS5P	1.00	+	1.00	-	-	-	Adhesive adenocarcinoma, with microinfiltration, plus normal alveoli.
13-1720BS2Ca	1.94	-	1.54	+	-	+	Invasive adenocarcinoma with extracellular mucus, mostly papillary adenocarcinoma, with few micro-papillary adenocarcinoma, grade II.
11-1433-9P	1.00	+	1.00	-	-	-	Lymph follicular hyperplasia
11-1433-2Ca	0.83	+	2.08	+++	+++	-	Squamous cell carcinoma, grade III.

*Score for percentage: no cell staining (-), 1-30% cells with weak staining (±), 1-30% cells with moderately positive staining (+), 30% cells with strongly positive staining or 30-60% cells with moderately positive staining (++), and > 60% cells with strongly positive staining (+++).

3). Supplemental Table S3. The top 4 functional networks of the differentially expressed genes in HIV-associated lung cancer.

ID	Top Diseases and Functions	Score	Focus Molecules	Molecules in Network
1	Cancer, Connective Tissue Disorders, Organismal Injury and Abnormalities	46	28	**ABI3BP**, **ALYREF**, **AUTS2**, Cbp/p300, **CBX3**, **CCDC102B**, Ck2, **CSNK2A1**, **ECT2**, estrogen receptor, **FHL1**, **FMNL2**, **GRK5**, Hsp90, **KDM5B**, **MEIS1**, **MYH10**, N-cor, **NEDD9**, **NIF3L1**, **NOL4L**, **PJA1**, **PURB**, **SCML4**, **SIX1**, **SLIRP**, **SMC4**, **SPTBN1**, **SRPK1**, **STIL**, thymidine kinase, **TOP2A**, **TTI1**, Vegf, **WDR12**
2	Nucleic Acid Metabolism, Small Molecule Biochemistry, Organ Morphology	41	26	26s Proteasome, **AGR2**, **ALDH18A1**, **AR**, **ARRB1**, **C6orf141**, **CCNB1**, **ENO1**, **FMO2**, **GART**, **GBAS**, Hdac, **HIST1H3H**, HISTONE, Histone h3, Hsp70, **HUS1**, **KIAA0895**, **NIPSNAP1**, **OSR1**, **PAICS**, **PCNA**, **PFAS**, Pka, RNA polymerase II, **RORA**, **SHMT2**, Smad, **SMARCA4**, **SS18L1**, **TARP**, **TARS**, trypsin, **WDR77**, **WHSC1**
3	Cardiovascular System Development and Function, Embryonic Development, Organismal Development	34	23	APC (complex), Cdc2, Cdk, Cyclin A, Cyclin D, Cyclin E, DUB, **DUOX1**, E2f, ERK, **HIST2H2BE**, **MAL**, **MED20**, **NFASC**, **PALB2**, **PRIM2**, **PSAT1**, **RAD51AP1**, Rb, **RECK**, **SCN4B**, **SCN7A**, **SLC19A1**, **TACC1**, **TBX2**, **TFAP2A**, **TFAP2C**, Tnf (family), **TOX3**, **TRIB3**, **TRMT13**, **USP21**, **USP22**, voltage-gated sodium channel, **ZNF326**
4	Cancer, Organismal Injury and Abnormalities, Developmental Disorder	32	22	**AKAP1**, **APBB2**, **CACNA1C**, Collagen Alpha1, **CREB3L4**, ERK1/2, **FOXC2**, **FOXF1**, **GDF10**, Gli, **GPC3**, Hedgehog, **HS6ST2**, **ITGA9**, **ITGB4**, **ITGB8**, **KCNN4**, Laminin1, **MUC5B**, **MYLK**, **PDE4D**, Pka catalytic subunit, Pkg, Pki, **PKIG**, Ppp2c, **RAB25**, **SEMA6A**, **SLC19A3**, SMAD1/5, Sox, **SOX9**, Tenascin, **VGLL3**, Wnt

*The genes labelled red were upregulated in HIV-associated lung cancer, the genes labelled blue were repressed in HIV-associated lung cancer, and the gens labelled black were unchanged in HIV-associated lung cancer.

4). **Supplemental Table S4.** The top 5 functional pathways of the differentially expressed genes in HIV associated lung cancer.

**Ingenuity Canonical Pathways**	**-log(p-value)**	**Ratio**	**Molecules**
Cellular Effects of Sildenafil	3.87E+00	7.75E-02	MYH10, KCNN4, PRKG1, PLCE1, SLC4A11, ADCY4, GUCY1A2, CACNA1C, MYLK, PDE4D
Dopamine-DARPP32 Feedback in cAMP Signaling	3.10E+00	6.21E-02	KCNJ8, PPP1R3D, PRKG1, PLCE1, PRKCQ, ADCY4, GUCY1A2, GNAQ, CACNA1C, CREB3L4
Purine Nucleotides De Novo Biosynthesis II	3.04E+00	2.73E-01	PAICS, PFAS, GART
5-aminoimidazole Ribonucleotide Biosynthesis I	3.00E+00	6.67E-01	PFAS, GART
Tetrahydrofolate Salvage from 5, 10- methenyltetrahydrofolate	2.49E+00	4.00E-01	MTHFD2L, GART

2. Supplementary Figures:

1). Supplemental Fig. S1. Partek analysis of microarray quality of our AIDS patients of lung cancer, the results show that our chips can distinguish cancer tissues and adjacent tissues. A) Source of variation analysis of HIV associated lung cancer; B) PCA analysis of 8 HIV lung tumor/adjacent normal tissue samples. Lung tumor tissue samples (Ca or Ca-1) labelled as red and blue; adjacent normal lung tissue samples (P，P-1) labelled as green and purple.

A **B**

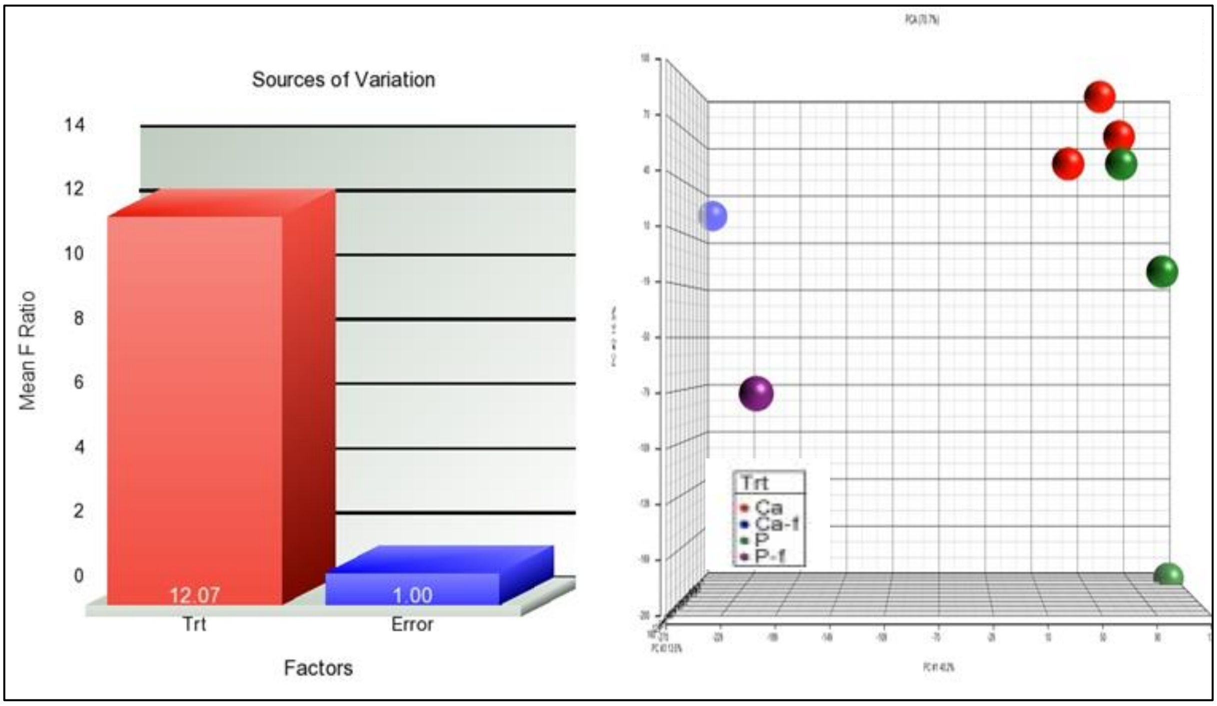

2). Supplemental Fig. S2. HE stains of lung HIV associated lung adenocarcinoma (Grade II) (A) and (Grade III) (B) tissue (original magnification ×200). Stains of invasive adenocarcinoma (Grade II) (C) and (Grade III) (D) tissue samples with an anti-SIX1 (original magnification ×400). The normal adjacent lung tissue was labelled with yellow arrows or tumor with red arrows.

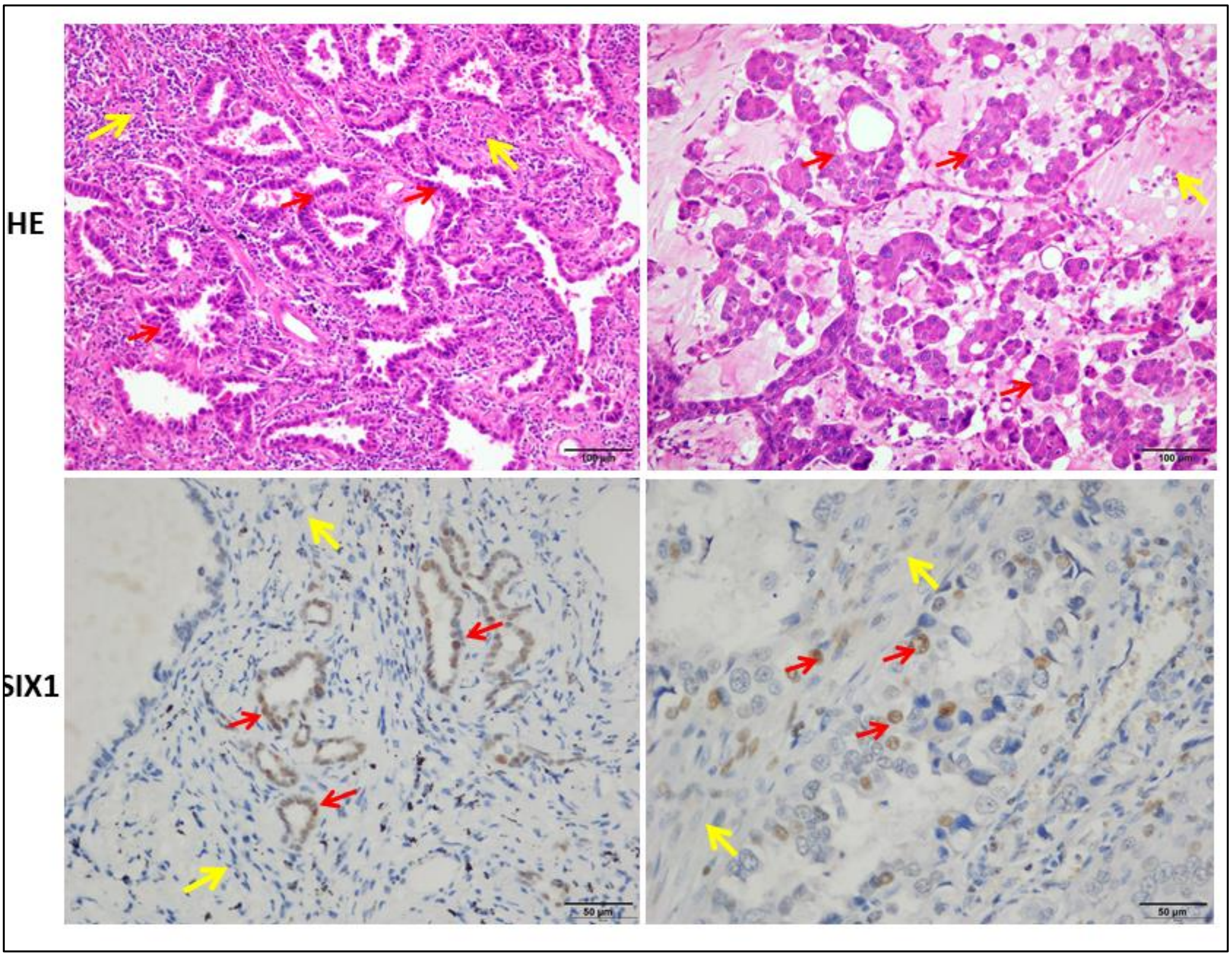

3) Supplemental Fig. S3. Functional network analysis of the differentially expressed genes in HIV associated lung cancer. A) cancer, connective tissue disorders, organismal injury and abnormalities; B) nucleic acid metabolism, small molecule biochemistry, organ morphology; C) cardiovascular system development and function, embryonic development, organismal development; D) cancer, organismal injury and abnormalities, developmental disorder

A

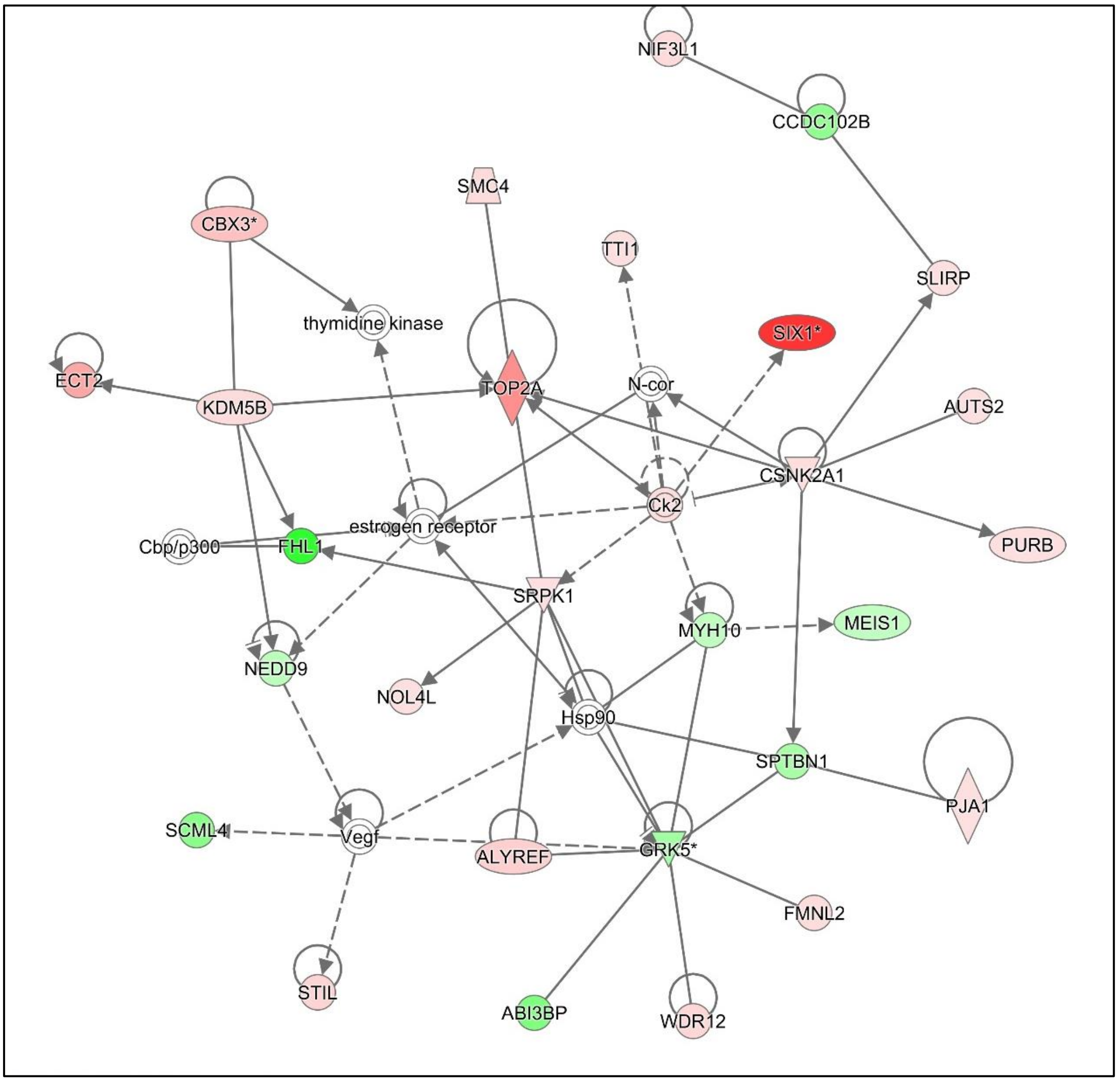

B

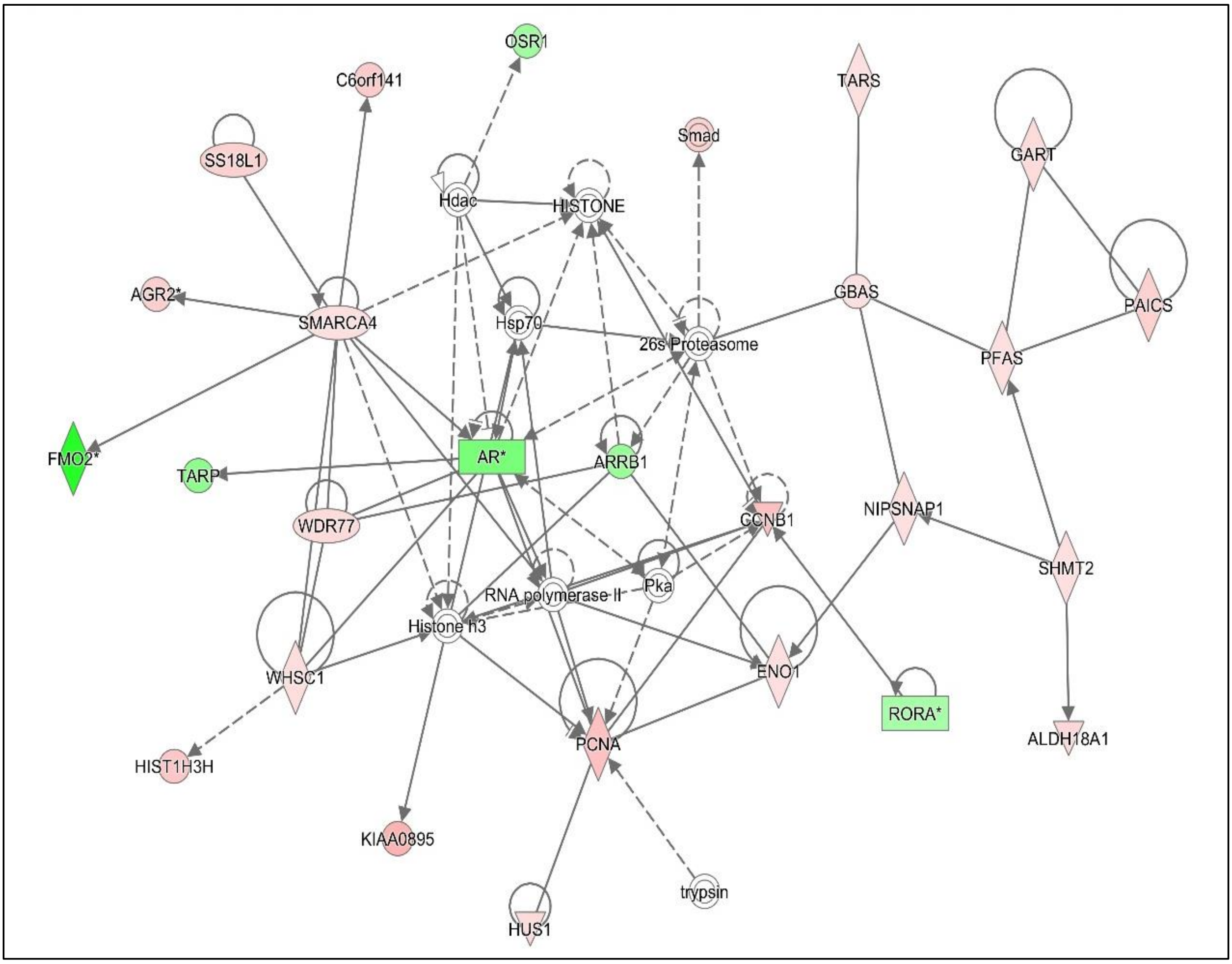

OSR1
C6orf141
TARS
Smad
SS18L1
GART
Hdac
HISTONE
AGR2*
GBAS
PAICS
SMARCA4
Hsp70
26s Proteasome
PFAS
FMO2*
AR*
ARRB1
TARP
NIPSNAP1
CCNB1
WDR77
SHMT2
RNA polymerase II
Pka
Histone h3
WHSC1
ENO1
RORA*
PCNA
ALDH18A1
HIST1H3H
KIAA0895
trypsin
HUS1

C

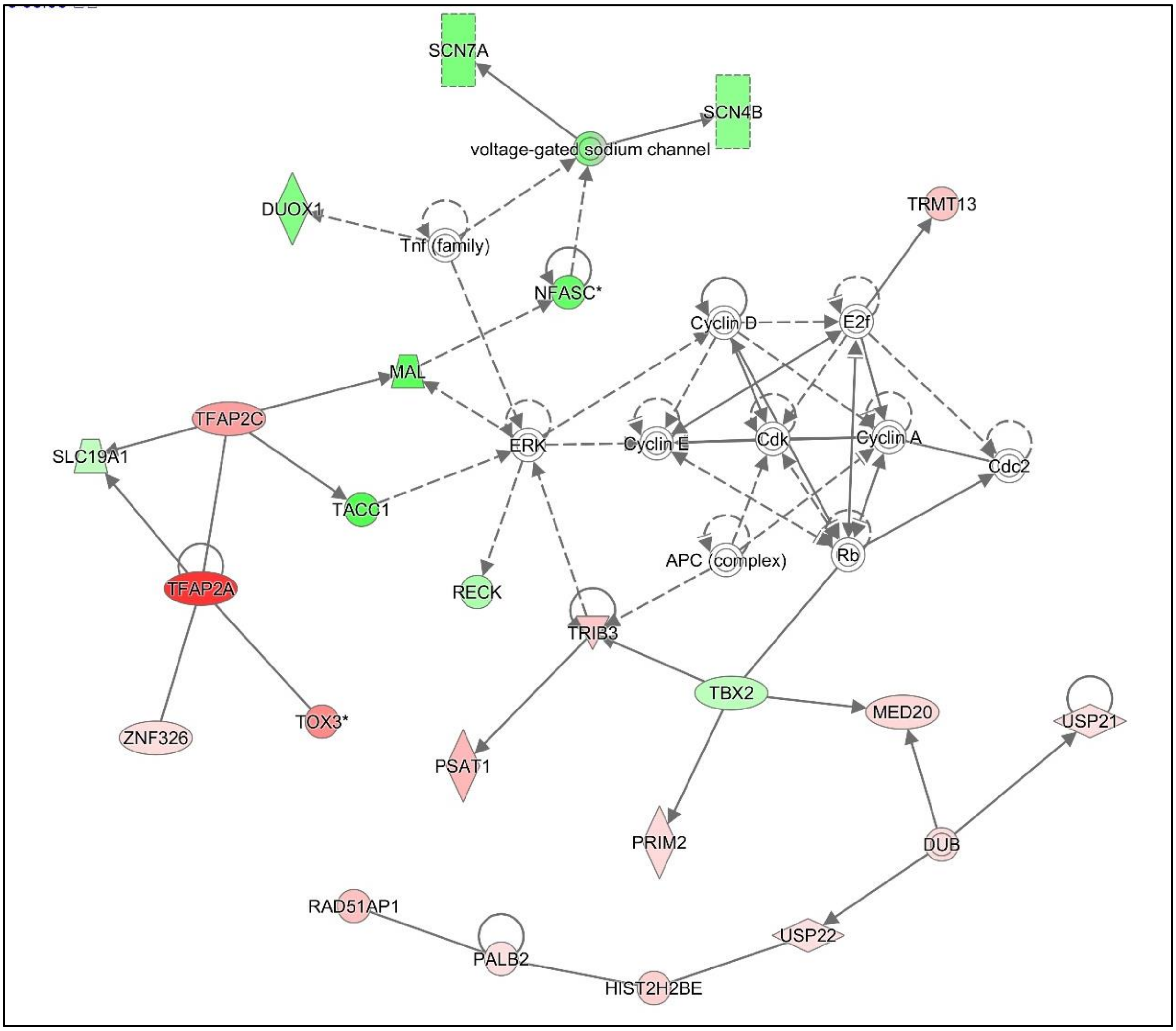
SCN7A
SCN4B
voltage-gated sodium channel
DUOX1
Tnf (family)
NFASC*
TRMT13
Cyclin D
E2f
MAL
TFAP2C
SLC19A1
ERK
Cyclin E
Cdk
Cyclin A
Cdc2
TACC1
APC (complex)
Rb
TFAP2A
RECK
TRIB3
TBX2
MED20
USP21
ZNF326
TOX3*
PSAT1
PRIM2
DUB
RAD51AP1
PALB2
USP22
HIST2H2BE

D

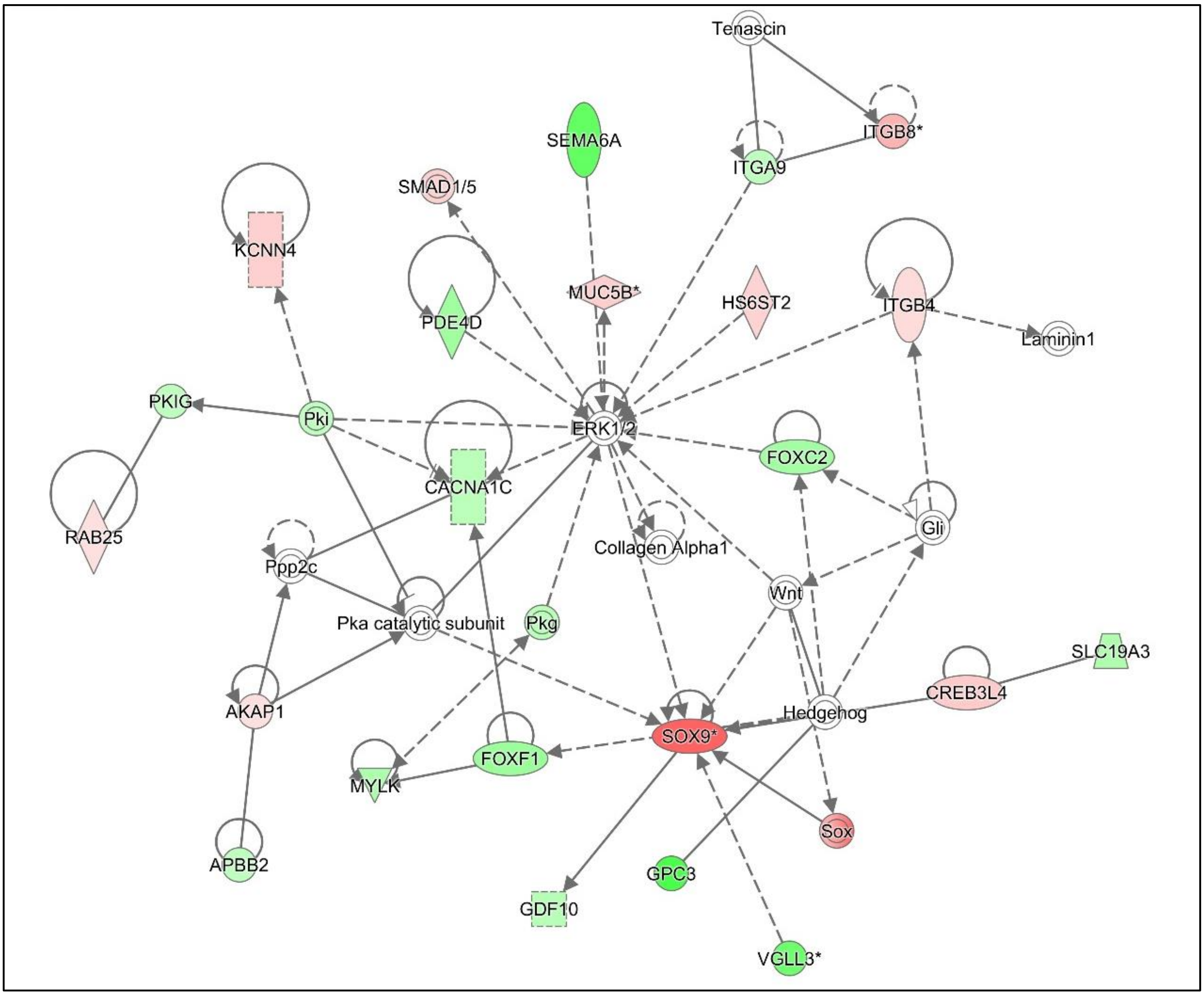
Tenascin
ITGB8*
SEMA6A
ITGA9
SMAD1/5
KCNN4
MUC5B*
HS6ST2
ITGB4
PDE4D
Laminin1
PKIG
Pki
ERK1/2
FOXC2
CACNA1C
RAB25
Collagen Alpha1
Gli
Ppp2c
Wnt
Pka catalytic subunit
Pkg
SLC19A3
CREB3L4
AKAP1
Hedgehog
SOX9*
FOXF1
MYLK
Sox
APBB2
GPC3
GDF10
VGLL3*
